# Drug Discovery Targeting Nicotinic Acetylcholine Receptors for Hearing Loss

**DOI:** 10.3390/ijms27083614

**Published:** 2026-04-18

**Authors:** Jordan Oman, Pooja Sapkota, Sameena Mateen, Marvin Schulte, Srinath Pashikanti

**Affiliations:** 1 Biomedical and Pharmaceutical Sciences, Idaho State University, Pocatello, ID 83209, USA; poojasapkota@isu.edu (P.S.); sameenamateen@isu.edu (S.M.); 2 Department of Pathobiology, Cummings School of Veterinary Medicine, Tufts University, North Grafton, MA 02155, USA; marvin.schulte@tufts.edu

**Keywords:** nicotinic cholinergic receptor, cochlea, Medial Olivocochlear (MOC) system, positive allosteric modulator (PAM), hearing loss

## Abstract

Hearing loss is detrimental to human health, and currently, more than 1.5 billion people are affected by hearing loss. Active military personnel and construction workers are examples of individuals in the workplace who are exposed to loud noise and are at serious risk of hearing loss. While there is currently no therapy for hearing loss, evidence supports investigating the enhancement of the Medial Olivocochlear (MOC) system, an efferent pathway for hearing that serves as a gain-control for hearing loss protection. Selectively modulating the α9α10 nicotinic acetylcholine receptor (nAChRs) found within this pathway is promising for the development of a new drug class. In this review article, we present the most current findings related to the therapeutic targeting of α9α10 nAChRs for hearing loss. We discuss the loss- or gain-of-function of the receptor, evaluate the known modulators of the receptor, examine their clinical relevance, and discuss their chemical and physical properties. Investigation of this novel pathway may aid in the development of a therapeutic for hearing loss.

## 1. Introduction

Hearing loss drastically impacts quality of life. It is estimated to affect approximately 1.5 billion people globally, with 430 million experiencing moderate to severe hearing loss [1]. Several factors can contribute to hearing loss. Some forms of hearing loss can be caused by age (presbycusis), genetics, and exposure to loud noise, leading to pathological changes in the auditory system.

Currently, there is a lack of therapeutic drugs for the prevention or improvement of hearing loss. Therapeutic targets of the auditory system are primarily located in the inner ear. Therapeutic strategies have focused on enhancing and restoring the structural and functional components of the cochlea, including the auditory nerve, spiral ganglion, inner and outer hair cells (OHCs), supporting cells of the Organ of Corti and the synaptic ribbon at the basal pole of the inner hair cells [2,3,4,5,6,7]. Recently, the Medial Olivocochlear (MOC) pathway, which regulates cochlear amplification, has been investigated as a pharmacological target due to its endogenous protective feedback mechanisms that reduce vulnerability to synaptopathy and support auditory synaptic function [8].

The MOC pathway is a feedback system that inhibits the transduction of sound in the cochlea through the auditory nerve [9] and contains a potential target, the α9α10 nicotinic acetylcholine receptor (nAChR), which mediates synaptic transmission between the MOC and OHCs [10,11]. MOC synaptopathy has been observed in both presbycusis [12,13] and noise exposure, leading to hidden hearing loss (HHL) [8].

Gain-of-function mutations in mice MOC α9α10 nAChRs have been shown to protect against noise-related hearing loss [8]. In this study, researchers tested three groups of mice. WT: wild type; Chrna9 KO: knock out; and Chrna9L9′T KI: gain of function. They measured mice’s auditory brainstem response (ABR) thresholds before acoustic trauma (AT) and then tested post-AT on days 1 and 7. WT mice showed increased ABR thresholds on day 1 but returned to baseline after 7 days. Knockout mice maintained high ABR thresholds even after 7 days. Gain-of-function mice showed no change after AT, thus preventing noise-induced hearing loss. Researchers also found that enhancing the MOC pathway not only prevented cochlear synaptopathy but also promoted new afferent synapse formation to occur after trauma. Beyond just acoustic overexposure, Maison et al. demonstrated that surgical de-efferentation accelerated age-related amplitude reduction in cochlear neural responses [14]. Boero et al. further this work on age-related hearing loss by demonstrating that MOC activation provided resistance to presbycusis [10]. The protective and regenerative mechanisms of the MOC system make its dual-action capabilities a therapeutic target for HHL by enhancing nerve fiber signaling and promoting synaptogenesis. By maintaining robust peripheral nerve signaling to the brain, other sensory neuronal disorders, tinnitus (abnormal CNS signals causing ringing), and hyperacusis (increased sound sensitivity) could be prevented.

The MOC system may be a promising target for treating hearing loss. Identifying modulators of the α9α10 receptor will help researchers develop novel analogs targeting this receptor. Investigation of the α9α10 nAChR within the MOC system may help prevent presbycusis, HHL, and noise-induced hearing loss.

## 2. Efferent Auditory System Is a Target for the Treatment of Hearing Loss

**Figure 1:**
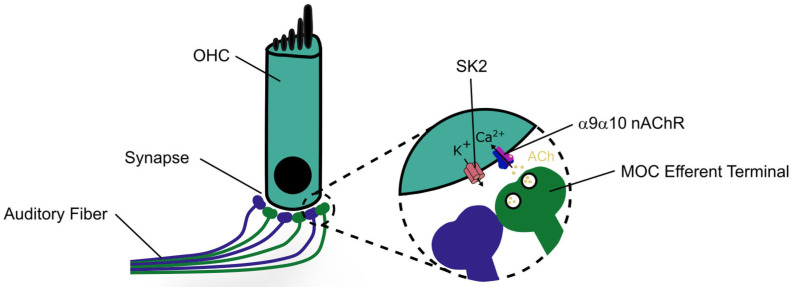
Cholinergic synapse of the outer hair cell (OHC) and auditory fiber. Efferent and afferent medial olivocochlear (MOC) fibers are shown in green and blue, respectively. The release of acetylcholine (ACh) activates α9α10 nicotinic acetylcholine receptors (nAChRs), leading to Ca^2+^ permeability and inhibition of the hair cell. The small-conductance calcium-activated potassium (SK2) channel is activated by calcium influx, driving hair cell hyperpolarization.

The role of α9α10 nicotinic acetylcholine receptors in hearing mechanisms is best understood from the study of the MOC pathway, which modulates the peripheral auditory system. The MOC system originates from the superior olivary complex and synapses with OHCs in the organ of Corti [10]. These synapses are mediated by α9α10 nAChRs (Figure 1), which increase intracellular Ca^2+^ levels of OHCs and lead to the opening of SK2 channels, thereby releasing K^+^ into the synapse and hyperpolarizing the cell [15]. This hyperpolarization serves as a protective feedback mechanism against auditory trauma by directly inhibiting cochlear amplification of sound [8]. Importantly, α9α10 receptors are primarily found within the ear [16] and are involved in regulating auditory processing [9]. Despite its established physiological roles, few pharmacological strategies have been applied to enhance α9α10 receptor protective mechanisms [11]; therefore, further investigation is warranted to understand its function and properties, and to develop small molecules that can target the receptor.

### 2.1. Unique Physiology of α9α10 nAChRs

The Cys-loop receptor superfamily comprises several pentameric cell-surface receptors that function as ligand-gated ion channels (LGICs), including those that respond selectively to neurotransmitters serotonin, GABA, glycine, glutamate, and acetylcholine (ACh) [17]. nAChRs are typically excitatory cationic channels within the nervous system [18]. nAChRs receptors are encoded by 17 genes in vertebrates, including 10α (α1–α10), 4β (β1–β4), γ, δ, and ε [19]. They were initially classified as muscle-type and neuronal-type based on their expression at postsynaptic neuromuscular junctions and neuronal cells. The muscle type exists as either (α1)_2_β1δε (adult receptor) or (α1)_2_β1δγ (fetal receptor). The alpha and beta subunits combine to form several potential molecular forms, such as homomeric α7 and α9 receptors and heteromeric α4β2 and α6β2 receptors, which exhibit distinct expression patterns and pharmacological properties [20]. These receptors can exist in different stoichiometric combinations, each with distinct pharmacological properties, including sensitivity to ACh, unique kinetic properties, and cation permeability [21]. The discovery of α9 and α10 subunits as a functional α9α10 ion channel in the non-neuronal mechanosensory hair cells further necessitated the expansion of the nicotinic receptor category as a hair cell receptor [22]. This is because hair cell receptors show significant differences in their expression and co-assembly rules compared to neuronal types: they coassemble only with each other, not with other nAChR subunits, and are only coordinately transcribed together in the inner ear [23]. The hair cell receptors were also found to exhibit sequence divergence from conventional nicotinic receptors, where the α9 and α10 subunits are most closely related to each other, followed by the α7-like subunits, reflecting their distinct evolutionary history, which could be a last common ancestor of Bilateria or possibly by an earlier split related to Eumetzoan and presenting with distinguishing pharmacological properties [18,23,24]. The assembly of α9 and α10 subunits could form a receptor with multiple stoichiometries. While the stoichiometries have been the subject of research, significant gaps remain in our understanding of the exact quaternary structures of α9α10 nAChRs. Evidence suggests that at least two stoichiometries, (α9)_2_(α10)_3_ and (α9)_3_(α10)_2_ are functional with three potential binding sites at α9(+)/α9(−), α9(+)/α10(−), and the α10(+)/α9(−) interface [25,26,27]. The orthosteric binding site is a conserved region at the extracellular domain of adjacent subunits. Loops A-C are found on the (+) face, while loops D-F are found on the (−) face [24,28]. While the putative stoichiometry for hair cells is (α9)_2_(α10)_3_ [25], multiple stoichiometries can complicate drug discovery, thereby affecting efficacy and potency.

### 2.2. Expression of α9 and α10 Subunits

The α9α10 heteromeric receptor is found predominantly expressed in cochlear and vestibular hair cells of the inner ear [29] and has also been reported in dorsal root ganglion neurons [30]. Although one study detected expression of the α9 and α10 subunits in the mouse brain [21], most published research indicates that the α9 and α10 subunits are absent from brain tissue [31]. Beyond just the nervous system, α9 and α10 subunits have been found in immune cells [32,33,34]. In addition, the α9 subunit has been implicated in lung [35,36] and breast [37,38,39] cancers.

Human α9 subunits and the α9α10 receptor complex exhibited limited translational efficiency in Xenopus laevis oocytes compared to their rat counterparts. A dramatic enhancement in receptor expression was achieved after the 5′ untranslated leader sequence from Alfalfa mosaic virus RNA4 (AMV leader) was supplemented to the 5′ leader sequence of α9 subunit cRNA. This modification boosted functional expression by 70-fold for α9 nAChRs and 80-fold for α9α10 nAChRs [40].

Functional homomeric α9 nAChRs have been shown to form in Xenopus oocytes using rat and human α9 subunits [29,41,42]. Mammalian α10 subunits do not form a functional homomer when expressed alone. However, α10 subunits can assemble as a functional receptor when co-expressed with α9 subunits, chaperone protein, resistance to inhibitors of cholinesterase-3 (RIC-3), or in the presence of exogenous alkaloids like strychnine, brucine, or methyllycaconitine [43,44].

While expression of the pentameric α9α10 receptor is challenging and has primarily been done in oocytes, it is also possible in cell lines, including the mammalian tsA201 [29] and the human embryonic kidney (HEK293T) cell lines. Assembly of the α9 and α10 subunits at the cell surface requires a cholinergic ligand such as ACh, and degradation of the ligand by acetylcholine esterase (AChE) abolishes surface expression. Beyond assembly and expression, non-AChR transmembrane components (TMIE and TMEM132e) are needed for activity; while these accessory proteins are not involved in α9α10 receptor assembly or trafficking, they are essential for α9α10 function [45]. Loss of α9α10 function can affect downstream components such as the SK2 channel, where inactivity can lead to olivocochlear fiber degeneration [46,47]. TMIE is essential for both mechanotransduction (MET) channels and α9α10 receptor function [45,48]. Mutations in TMIE have been implicated in underlying nonsyndromic human deafness, which causes structural damage to hair cells and profound deafness [49]. These discoveries not only provide novel tools for studying the α9α10 on hair cells in cell lines but also identify peripheral auditory therapeutic targets, such as regulating TMIE gene expression, inhibiting AChE to promote α9α10 expression, and releasing Ca^2+^ to activate SK2 channels.

Screening in an animal model is an important consideration in the drug discovery process. While Zebrafish are commonly used to study ototoxicity and hearing loss, they exhibit enriched expression of the α9 subunit rather than the α10 [50], making them less ideal than other animal models unless genetically modified. α9 and α10 subunits are expressed in mice [51], as well as in rats [42,52]. Other species, such as non-human primates, may have even more closely related physiology for α9α10 receptors compared to humans, due to highly conserved genetics and epigenetics [53].

### 2.3. Modification of α9α10 nAChRs

In addition to the studies by Boero demonstrating enhancement of the MOC system with gain-of-function knock-in mice, which showed auditory protection and prevention of HHL, other gain-of-function experiments, such as those by Slika, have shown similar results. α9L9′T-HA-injected C57BL/6J wild-type mice were tested against wild-type and enhanced green fluorescent protein (GFP) control groups, and were exposed to noise at 5 weeks old. All groups were exposed to an octave-band (8–16 kHz) stimulus at 100 dB for 1 h, which can cause irreversible synaptic damage, and post-AT gain-of-function α9L9′T-HA group showed significantly lowered ABR absolute values and threshold shifts compared to control groups [54]. Showing protection against noise-induced trauma.

However, Lauer’s results contrast with these examples of MOC feedback contributing to hearing loss. Where α9 nAChR knockout (α9KO) mice showed little effect on age-related and noise-induced hearing loss. ABR thresholds and wave amplitudes were measured for age groups of 1–5 months, 6–10 months, and 11–15 months. α9KO and wild-type mice were tested in a noisy and quiet environment for each experiment. The results showed no accelerated progression of hearing loss in α9KO mice, and the noisy environment did not accelerate hearing loss in these mice [55]. However, these noise paradigms were quite different. In Lauer’s previously characterized noisy environment, sound levels ranged from 73.04 to 111.05 dB, with an average around 80 dB during workday periods. Additional transient sounds generated by researchers or staff could only briefly exceed 100 dB and then return to baseline [56]. In contrast, Boero and Slika groups used an acute trauma model in which mice were exposed to continuous stimuli (8–16 kHz, 100 dB) for 1 h [8,54]. These periods of intermittent noise exposure allow partial metabolic recovery of cochlear hair cells [57], whereas continuous stimulus does not. Further investigation of this pathway is warranted, given that gain-of-function assays show promise as a valid therapeutic target for AT.

## 3. Pharmacology of α9α10 nAChRs Potentiators

Agonists of the α9α10 receptor elicit a biological response, causing the opening of the LGIC on the postsynaptic OHC. Calcium enters the cell, activating SK2 channels that release potassium, causing hyperpolarization of the cell. This hyperpolarization inhibits OHCs, thereby reducing the auditory stimulus from the afferent pathway [58]. Agonists for the α9α10 receptor are listed in Table 1. The neurotransmitter for the α9α10 receptor is ACh and has been tested in human, rat, and chicken nAChRs with similar results [42,59,60]. Recently, diEPP piperazine cationic scaffolds have been identified as agonists of α9α10 [60]. PhDu and CF_3_PhDu show greater responses than ACh, making them full agonists. This has led to the identification of a selective agonist for the receptor and will aid in a greater understanding of the receptor and its function in various diseases. Partial agonists of the α9α10 receptor act similarly to agonists; however, activation of the receptor with these ligands only results in partial efficacy. Efficacy is compared to the endogenous ligand ACh, which has 100% efficacy [16,60]. Partial agonists have a wide range of efficacies, which is beneficial in stimulating the receptor to a certain degree. Interestingly, oxotremorine, a selective muscarinic receptor agonist, acts as a partial agonist at α9α10 receptors, with very low efficacy due to the receptor’s atypical pharmacology [16].

**Table 1:** Potentiators of α9α10 nAChRs.

Agonist/ Partial Agonists	Structure	α9 EC_50_	E_Max_	α9α10 EC_50_	E_Max_	Refs.
Acetylcholine (ACh)	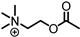	^A^ 26 ± 4 μM	1.00 ± 0.04	^A^ 30 ± 10 μM	#	[59,60]
		^B^ 12.9 ± 1.1 μM	Full agonist	^B^ 17.1 ± 3.1 μM	#	
		^C^ 12.9 ± 1.4 μM	Full agonist	^C^ 13.1 ± 2.6 μM	#	
Choline	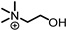	^B^ 188 ± 40 μM	0.87 ± 0.06	^B^ 541 ± 62 μM	0.38 ± 0.03	[59]
		^C^ 280 ± 20 μM	0.74 ± 0.06	^C^ 200 ± 16 μM	0.88 ± 0.07	
Carbachol	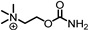	^B^ 64 μM	Na	^B^ 159 ± 31.5 μM	0.76 ± 0.05	[16,59]
				^C^ 150 ± 15 μM	0.90 ± 0.07	
p-CONH	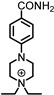	^A^ 1.15 ± 0.25 μM	0.80 ± 0.04	#	#	[60]
p-CN	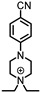	^A^ 0.368 ± 0.10 μM	0.76 ± 0.05	#	#	[60]
PhDu	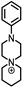	^A^ 2.38 ± 1.52 μM	1.69 ± 0.22	#	#	[60]
CF_3_PhDu	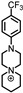	^A^ 0.475 ± 0.22 μM	1.38 ± 0.10	#	#	[60]
p-CF_3_	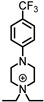	^A^ 7.04 ± 1.88 μM	0.36 ± 0.02	#	#	[60]
DMPP	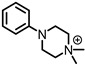	^B^ Na	Partial agonist	^B^ 21.5 ± 4.0 μM	0.006 ± 0.003	[16,59]
				^C^ 9.8 ± 0.5 μM	0.32 ± 0.03	
Oxotremorine	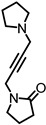	^B^ Na	Partial agonist	Na	0.01 Partial agonist	[16]

Potentiators of α9α10 and α9 are listed with EC_50_ (half-maximal effective concentration) and E_Max_ (maximal efficacy). #—means the results from the α9α10 assay are similar to α9 data shown. Na—signifies data not known. ^A^ Human nAChR, ^B^ Rat nAChR, ^C^ Chicken nAChR. Compounds with E_Max_ ≥ 1.00 are full agonists.

## 4. Atypical Pharmacology and Inhibition of α9α10 nAChRs

Heteromeric α9α10 nAChRs are the atypical members of the neuronal nicotinic receptor family; while nicotine generally agonizes nAChRs, it antagonizes α9α10 nAChRs (Table 2) [41,61]. α9α10 pharmacological characteristics differ not only from neuronal nAChRs but also from muscle-type nAChRs, muscarinic agonists like pilocarpine, and muscarine, which typically act as agonists, acted as antagonists [62]. Other non-nicotinic ligands, such as strychnine (a glycine receptor antagonist) and bicuculline (a GABAA receptor antagonist), have high binding affinities to these receptors [29]. Owing to the peculiar pharmacological feature of α9α10 nAChRs, a better understanding of these receptors could benefit the prevention of several diseases mediated by nicotinic acetylcholine receptors, such as Alzheimer’s and Parkinson’s diseases, schizophrenia, anxiety, depression, and pain [40,42]. Antagonists have been investigated for the treatment of pain with promising results [63,64,65]. While nicotine use has been associated with hearing loss in clinical studies [66,67], some studies suggest that nicotine can improve tone-in-noise detection, leading to enhanced auditory gating function [68]. Further investigation is needed to understand how inhibition of this receptor contributes to AT.

**Table 2:** Inhibitors of α9α10 nAChRs.

Antagonist	Structure	α9 IC_50_	Agonist	α9α10 IC_50_	Agonist	Refs.
Nicotine	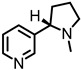	^A^ 31.5 μM	ACh	^A^ 46.3 ± 12.4 μM ^B^ 39.4 ± 3.4 μM	ACh	[16,59]
d-tubocurarine	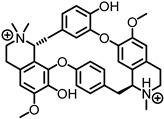	^A^ 300 nM	ACh	^A^ 110 nM	ACh	[16]
α-bungarotoxin	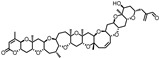	^A^ nM range	ACh	^A^ nM range	ACh	[16]
Atropine	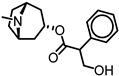	^A^ 1 μM	ACh	^A^ 1 μM	ACh	[16]
Muscarine	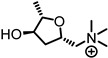	^A^ 75–83 μM	ACh	^A^ 41 μM	ACh	[16]
Strychnine	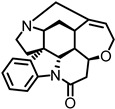	^A^ 18 nM	ACh	^A^ 20 nM	ACh	[16]
Bicuculline	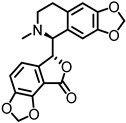	^A^ 0.8 μM	ACh	^A^ 1 μM	ACh	[16]
Tropisetron, ICS-205,930	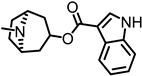	^A^ 20 nM	methiodide	^A^ 20 nM	methiodide	[16]

Inhibitors of α9α10 and α9 are listed with IC_50_ (half-maximal inhibitory concentration) and agonist used. ^A^ Rat nAChR, ^B^ Chicken nAChR.

## 5. Enhancement of α9α10 nAChRs Activity with Positive Allosteric Modulators

Positive allosteric modulators (PAMs) act by increasing the efficacy and/or potency of the endogenous ligand, while having no effect by themselves. Type I PAMs increase the efficacy or maximal response of the orthosteric ligand without altering EC_50_, while Type II PAMs can lower the EC_50_ [69]. Currently, only two PAMs for the α9α10 receptor have been identified: L-ascorbic acid (Vitamin C) and Ryanodine [61,70]. Both of these ligands are water-soluble, polyhydroxylated scaffolds. L-ascorbic acid significantly increases the efficacy more than two-fold [61]. Shown in Table 3 is the ACh EC_50_ for L-ascorbic acid and Ryanodine. Ryanodine is more potent (200 µM) but does not increase efficacy (1.36) as much as L-ascorbic acid (3 mM, 2.40) [61,70]. L-ascorbic acid’s activity as an antioxidant, cofactor, and enzyme is typically attributed to its enediol moiety, which makes it very acidic because of the delocalized system. Discovery of additional PAMs for this receptor could lead to new therapeutics for hearing loss.

**Table 3:** Positive Allosteric Modulators (PAMs) of α9α10 nAChRs.

PAM	Structure	α9 ACh EC_50_	E_Max_	α9α10 ACh EC_50_	E_Max_	Ref.
L-ascorbic acid (3 mM)	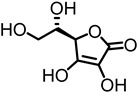	Na	Na	^A^ 18 ± 1 µM ACh	2.40 ± 0.20	[61]
Ryanodine (200 µM)	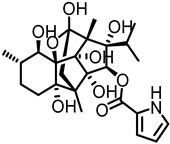	Na	Na	^A^ 15.4 ± 1.63 µM ACh	1.36 ± 0.13	[70]

Potentiators of α9α10 and α9 are listed with EC_50_ (half-maximal effective concentration) and E_Max_ (maximal efficacy). Na—signifies data not known. ^A^ Rat nAChR.

## 6. Clinical Relevance of Potentiators/Inhibitors of α9α10 nAChRs

The molecules listed in Table 1, Table 2 and Table 3 are clinically relevant or used as research tools. The clinical relevance of select molecules is discussed. Understanding the effects of these molecules can aid in drug repurposing and drug development, helping avoid unwanted targeting.

### 6.1. Multifunctional Actions of Known α9α10 nAChRs Agonists

Cholinergic drugs act similarly to ACh by mimicking its effects, activating both nicotinic and muscarinic receptors. Choline is a precursor to ACh and plays a vital role in the synthesis of phospholipids (sphingomyelin and phosphatidylcholine) [71]. The ammonium cation head group of choline is essential for its activity, and this activity has been investigated in cognitive disorders, such as dementia and Alzheimer’s disease [72], as well as in movement disorders, including tardive dyskinesia [73]. Carbachol is a cationic ammonium salt and carbamate ester used in the treatment of glaucoma. It activates M3 muscarinic receptors, causing pupil constriction, which increases the aqueous humor outflow and reduces pressure [74]. Oxotremorine is a muscarinic acetylcholine receptor (mAChR) agonist that is commonly used to study Parkinson’s disease by inducing tremors [75]. It is also used to study Alzheimer’s, hypertension, and depression [76,77,78,79].

### 6.2. Multifunctional Actions of Known α9α10 nAChRs Antagonists

Extra neuronal expression of α9 receptors can trigger metabotropic pathways. In immune cells, α9-containing nAChRs are involved in modulating pain signaling pathways, regulating inflammation, promoting cancer cell proliferation and metastases, and even inhibiting apoptosis [24,80]. This diverse expression of α9-containing nAChRs expands the therapeutic potential of targeting them, particularly through the development of selective inhibitory ligands aimed at treating chronic pain, inflammation, and certain types of cancers.

Nicotine is a dibasic compound that is monoprotonated at physiological pH, giving it a cationic charge. Nicotine displays atypical subtype-dependent pharmacology, activating many neuronal nAChRs while showing inhibitory actions at α9α10 receptors. While its clinical relevance is related to its substance use dependence, and risks of cardiovascular disease and cancer [81,82], there is research into its effects on Parkinson’s disease, Alzheimer’s disease, and hearing loss [68,83,84,85]. D-tubocurarine, a bis-quaternary alkaloid, was historically used as the first non-depolarizing neuromuscular blocking agent [86]. It binds at the α-γ and α-δ interfaces of the muscle-type nAChR, acting as a reversible competitive antagonist, which leads to muscle relaxation and paralysis [87]. Atropine is an antagonist of mAChRs and nAChRs that is clinically used to treat bradycardia, organophosphorus, and carbamate poisoning [88]. The ester of atropine is vital for binding to the active site and blocking the binding of ACh [89].

Venom-derived peptides, such as highly selective α-conotoxin (including Vc1.1, RgIA, PeIA, GeXIVA) from cone snails and less selective three-finger α-neurotoxins (α-Bungarotoxin, α-cobratoxin) from cobra venoms, are potent inhibitors of α9α10 nAChRs [90]. The analgesic effect of these peptides in neuropathic pain models suggests a non-opioid alternative for pain management [63].

### 6.3. Multifunctional Actions of Known α9α10 nAChRs PAMs

There are only two PAMs for α9α10 nAChRs. L-ascorbic acid is well known for its roles as an antioxidant, radical scavenger, and cofactor [91,92]. Its enediol moiety has been associated with its biological activity [93]. Clinically, it has been used to treat vitamin deficiency, including scurvy, to promote wound healing of cuts and burns, and to stimulate the immune system to fight the common cold and other infectious diseases such as tuberculosis [94,95,96,97]. It has been investigated for its anticancer, cardiovascular disease, and age-related disease activity [98,99,100]. Ryanodine, on the other hand, is primarily used as a research tool to investigate calcium signaling of sarcoplasmic reticulum and endoplasmic reticulum [101]. The pyrrole-2-carboxylate ester is critical for binding to ryanodine receptors to release calcium [102].

## 7. Desensitization of α9α10 nAChRs

The receptor kinetics of nAChRs are important for potentiation mechanisms. nAChRs generally have three conformational states: closed, open, and desensitized [103]. In the closed state, agonist is unbound or weakly bound without the channel opening. Upon sufficient agonist binding, the receptor transitions to the open state, allowing ion flux through the channel within milliseconds. Desensitization occurs when the channel transitions to a nonconducting state despite the presence of bound agonist. Prolonged or repetitive agonist stimulation further promotes desensitization, leading to the inactivation of the ion channel [104]. One of the conventional roles of desensitization is thought to be the prevention of excessive receptor stimulation since nAChRs are permeable to calcium and can thus be potentially excitotoxic to neurons [105].

The transition of nAChRs from the open-channel conformation to the desensitized state determines the duration and intensity of agonist-induced responses [106]. This can critically affect the neuronal signaling mechanism, as seen with nicotine. The prolonged inactivation of nAChRs following the nicotine exposure and slower recovery rate leads to nicotine addiction, tolerance, and dependence [107]. The susceptibility of receptors to desensitization depends on subunit arrangement and their possible stoichiometries and can affect their molecular properties and physiological functions of the receptors [108,109]. However, since desensitization is primarily associated with orthosteric ligands, desensitization can be modified by modulators. Allosteric modulators can enhance endogenous neurotransmission without directly stimulating nAChRs, offering greater subtype selectivity and avoiding receptor desensitization, especially Type II PAMs [110].

Loss of receptors can be problematic, as repetitive exposure to an agonist can lead to enhanced desensitization as a protective mechanism. This causes synaptic changes attributed to aging, exposure to loud noise, and ototoxic drugs, which can disrupt the cellular redox balance of hair cells’ mitochondria, leading to DNA damage and hair cell apoptosis [111]. In such cases, PAMs of α9α10 nAChRs can compensate for the loss of receptors in the cochlear OHCs, protecting hearing function [11].

## 8. Anatomical and Structural Barriers to Inner Ear Drug Delivery

### 8.1. Physical and Biological Obstacles to Inner-Ear Drug Delivery

**Figure 2:**
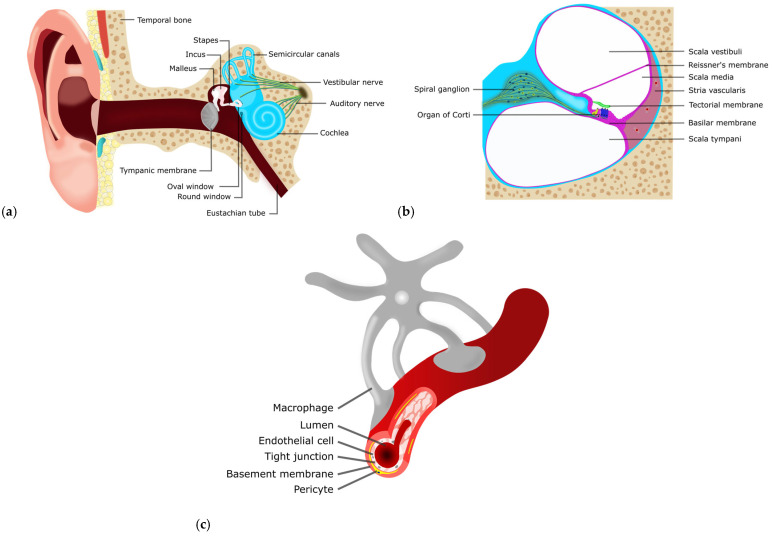
Overview of the ear and its anatomical barriers. (**a**) Anatomy of the ear showing components of the middle and inner ear. Membranes, such as the tympanic membrane (gray), oval window (OW), and round window (RW), play a role in drug diffusion and permeability. (**b**) Cross-section of the cochlea. This shows the three unique chambers within the cochlea. The scala vestibuli (upper chamber) and scala tympani (bottom chamber) are full of perilymph (negative electrical potential), while the scala media (middle chamber) is full of endolymph (positive electrical potential). Additional membranes separate the fluid contained within these unique chambers. The organ of Corti, located within the scala media, houses the outer (blue) and inner (yellow) sensory hair cells that convert mechanical vibrations into electrical signals. The stria vascularis (mauve) plays a crucial role in generating the endocochlear potential and facilitating the transport of nutrients into the inner ear from the bloodstream. (**c**) The blood-labyrinth barrier (BLB) found within the stria vascularis plays a role in the movement of substances into the cochlea from the bloodstream and is essential for normal hearing function.

Potential therapeutic drugs targeting inner-ear moieties are challenging to deliver, prompting the development of new strategies and methods. Inner ear drug delivery is challenging due to anatomical barriers, beginning with its location within the petrous portion of the temporal bone (Figure 2a). This bony labyrinth is the densest bone in the human body due to the high amount of hydroxyapatite [112], which restricts pharmacological access.

### 8.2. Challenges with Local Delivery to the Inner Ear

Even when drugs are locally delivered, additional anatomical barriers must be overcome. Membranes such as the tympanic membrane (eardrum), round window (RW), and oval window (OW) (Figure 2a) limit drug delivery into the cochlea. Intratympanic (IT) injections release drugs directly into the middle ear and diffuse across the OW or RW into the perilymph, which then must cross additional membranes to enter the scala media, where the drug can reach the organ of Corti (Figure 2b). A small incision on the tympanic membrane (myringotomy) can be used for drug injections into the inner ear. Microcatheters can also be surgically placed, bypassing membrane barriers, to deliver drugs repeatedly directly into the middle or inner ear. Drug delivery into the middle ear can be eliminated through the eustachian tube into the nasopharynx, thereby significantly impacting the overall pharmacokinetics [113].

Intracochlear delivery bypasses middle-ear anatomical barriers by direct injection into the cochlea and avoids inconsistent diffusion across the RW by delivering directly into the scala tympani. This requires a surgical procedure, such as a cochleostomy, to insert an intracochlear electrode array. While this maximizes drug bioavailability, the drilling process poses a significant risk to delicate inner ear structures, which can affect residual hearing [114].

### 8.3. Challenges with Systemic Delivery to the Inner Ear

Because local and surgical routes pose increased risks, minimally invasive routes are attractive alternatives. However, systemic delivery faces its own challenges due to the blood-labyrinth barrier (BLB) once in circulation. The BLB is similar to the blood–brain barrier (BBB) in that it regulates the movement of substances. It separates fluids, specifically within the stria vascularis interstitial space and the bloodstream [115]. The BLB (Figure 2c) is composed of similar components to the BBB, which include endothelial cells, tight junctions, basement membrane, and pericytes; however, the BLB additionally has perivascular resident macrophage-like melanocytes (PVMs/M), which contribute to barrier permeability, integrity, endocochlear potential, maintenance, and repair [116].

To overcome both local and systemic delivery limitations, non-invasive approaches such as nanoparticles, hydrogels, and chemical permeation enhancers are being developed to directly facilitate membrane passage and increase drug permeability [117,118,119]. Drug delivery into the Organ of Corti is vital because the MOC system synapses at the OHCs, where the α9α10 nAChRs are located.

## 9. Drug Permeability into the Inner Ear Beyond the Rule of Five

Lipinski’s Rule of Five is a widely accepted set of guidelines for predicting the oral bioavailability and passive diffusion of small molecules [120]. Compounds that obey this Rule are more likely to permeate biological membranes, such as the RW membrane [121]. However, with systemic delivery into the inner ear, many ototoxic agents (e.g., aminoglycosides, cisplatin) violate these rules yet still access the inner ear through the BLB. This suggests that alternative mechanisms beyond passive diffusion govern cochlear entry for many therapeutic and toxic compounds [122]. The Rule of Five is crucial in predicting oral bioavailability and membrane permeability. Compounds following these rules are more likely to be membrane-permeable:

- Molecular weight (MW) ≤ 500 Da [120];
- LogP ≤ 5;
- H-bond donors (HBD) ≤ 5;
- H-bond acceptors (HBA) ≤ 10;
- Number of rotatable bonds (RB) ≤ 10 [123].

However, there are exceptions to the Rule, and compounds that violate the Rule of 5 may be important when considering drug candidates (e.g., cross the BLB into the endolymph) [124]. Table 4 shows the physicochemical properties of various compound classes that may be important to consider for drug development into the inner ear. These classes consist of nicotinic modulators, aminoglycosides, platinum drugs, steroids, and loop diuretics. Nicotinic modulators are small molecules that are often lipophilic and typically diffuse passively. Aminoglycosides are large polar molecules that readily dissolve in water and tend to require ion channels, transporters, or pores for entry [125,126]. Platin-based drugs are small molecules with moderate molecular weights (MWs), where the central platinum neutralizes the molecule. Steroids typically undergo simple diffusion into cells; however, recent studies show that this may not always be the case [127]. Lastly, loop diuretics are small molecules that can cross membranes and alter ion concentrations of inner ear fluids [128]. Ionic diffusion may play a crucial role due to the selective permeability of inner ear compartments. Further research identifying channels and carriers that facilitate the transport of charged small molecules could aid the development of novel therapeutics targeting the inner ear. Select compounds from these classes have been further analyzed to identify ideal drug candidates.

**Table 4:** Summary of Lipinski’s Rule of Five and cochlear entry.

Compound Class	MW	LogP	HBD	HBA	Cochlear Entry	Lipinski Violation	Transport
Nicotinic modulators	≤500	≤5	≤5	≤10	Passive diffusion	Rare	No
Aminoglycosides	>500	<0	>5	>10	Ion channels, transporters, or pores	Yes (2–3)	Yes
Platinum drugs	~300	Low	2–4	4–8	OCT transporters	Maybe	Yes
Steroids	~390	1–3	1–3	4–6	Passive diffusion	No	Sometimes
Loop Diuretics	~300	1–4	1–2	4–6	Passive diffusion	Rarely	Sometimes

Table 5 compares compounds from each class based on their chemical properties. Properties include MW, LogP, HBD, HBA, RB, violations of Lipinski’s Rule of 5, entry into cochlea, and other characteristics. Another essential characteristic is the ionization state of the compounds at physiological pH; many amine-containing compounds can be protonated, resulting in a cationic state. Many of the drugs designed for hearing treatment have poor cochlear distribution, with higher LogP and lower topological polar surface area (TPSA) [124].

**Table 5:** Physicochemical properties of compounds that enter the inner ear.

Compound Class	Compounds	MW (Da)	LogP	HBD	HBA	RB	Lipinski Violations
Aminoglycosides	Gentamicin	477.6	−3.1	8	12	7	2
	Tobramycin	467.5	−5.8	10	14	6	2
	Netilmicin	475.6	−3	8	12	8	2
Platinum drugs	Cisplatin	300.1	−2.2	2	2	0	0
Steroids	Dexamethasone	392.5	1.9	3	6	2	0
	Hydrocortisone	362.5	1.6	3	5	2	0
	Methylprednisolone	374.5	1.9	3	5	2	0
Loop Diuretics	Furosemide	330.7	2.0	3	7	5	0
	Bumetanide	364.4	2.6	3	7	8	0

Studies using guinea pigs have provided data about the distribution of the drugs from Table 5 in Table 6 in the perilymph and endolymph fluids using oral and injection delivery methods [129,130,131,132,133]. Distribution of steroids directly injected into the middle ear resulted in the highest concentration in inner ear fluids, whereas intravenous (IV) administration produced almost negligible levels. Platinum drugs and loop diuretics showed similar concentrations after a short time. Aminoglycosides showed similar concentrations to platinum drugs and loop diuretics when given intravenously, but after a much longer time period, even with violating Lipinski’s Rule of Five. Further studies are needed to determine the distribution of nAChR agonists and antagonists in the inner ear compartments, thereby predicting drug efficacy, interactions, and side effects. BLB-on-a-chip screening tools are currently being developed that try to mimic the permeability and integrity of the stria vascularis [134]. Understanding the physicochemical and pharmacokinetic properties of these drug classes could further improve the development of more accurate screening tools for the inner ear.

**Table 6:** Pharmacokinetic properties of compounds that enter the inner ear.

Compound Delivery Dose (mg/kg)	Time After Administration		Highest Concentration (mg/L)		Cochlear Entry Mechanism	Other Characteristics	Refs.
	Peri-Lymph	Endo-Lymph	Peri-Lymph	Endo-Lymph			
Gentamicin IV 40	2 h		8.05		Trans-strial trafficking	Ototoxic; accumulates in hair cells via MET channels	[126,129,135,136]
Tobramycin IV 40	4 h		6.78		Similar to gentamicin	Hair cell toxicant	[129,135]
Netilmicin IV 40	1 h		4.17		Similar to gentamicin	Ototoxic	[129,137]
Cisplatin IV 12.5	20 min		4.2		Passive diffusion	ROS-mediated ototoxicity	[122,130]
Dexamethasone					Passive + GR-mediated uptake	Anti-inflammatory	[121,131]
IT	1 h	1 h	1.5 ± 1.2 ^†^	9.1 ± 5.5			
IV	0 h		0				
Oral	0 h		0				
0.2							
Hydrocortisone					Passive diffusion	Anti-inflammatory and immunosuppressant	[131]
IT	1 h	2 h	72.4 ± 23.3	195.3 ± 80.6			
IV	1 h		0.23 ± 0.05				
Oral	2 h		0.09 ± 0.01				
4							
Methylprednisolone					Passive diffusion	Sudden SNHL therapy	[122,132,138]
IT	1 h	2 h	50.4 ± 19.1	186.0 ± 73.4			
IV	2 h		0.08 ± 0.05				
Oral	4 h		0.01 ± 0.02				
4							
Furosemide IV 100	15 min	1 h	4.9	1.6	Passive diffusion	Alters ion homeostasis	[133,139]
Bumetanide IV 30	~1 min		1.9		Passive diffusion	Experimental use	[134]

All previous studies tested compounds in guinea pigs. ^†^ Average of scala tympani and scala vestibuli. Intravenous (IV) and intratympanic (IT).

## 10. Conclusions

The growing public health challenge of hearing loss, related to noise exposure and age, significantly impacts quality of life and necessitates a reconceptualization of auditory protection. This review highlighted α9α10 nAChRs as an exceptional candidate due to their selectivity, atypical pharmacology, and physiology [25,61,62]. Their activation promotes synaptogenesis and helps preserve high-fidelity synaptic connections [8].

A critical evaluation of the current literature reveals a functional disparity; while knockout α9 studies showed no significant effect on hearing loss [55], gain-of-function studies demonstrated prevention of hearing loss and promoted synaptogenesis [8,54,140]. Pharmacological results for this pathway are still preliminary. Currently, there are a limited number of selective ligands for the receptor, and unlike genetic models that can continuously enhance pathways, ligand activation of these pathways is limited by receptor desensitization kinetics [141]. Designing a selective PAM addresses pharmacological gaps by avoiding desensitization and enhancing therapeutic effects.

Identification of a selective PAM is only part of the solution; permeability and potency are equally important. Our analysis of the physicochemical landscape (Table 5) identified properties that help molecules diffuse into the cochlea’s apical regions, such as lower LogP, increased HBD/HBAs, and RBs, while maintaining potency. We propose that future drug design incorporate higher TPSA and specific ionization states to overcome BLB permeability.

In summary, we posit that designing a potent PAM for the α9α10 nAChR could address the root cause of maladaptive central gain by targeting both HHL and phantom signaling in tinnitus and hyperacusis related to noise- and age-related hearing loss. Accordingly, further investigation of this pathway will advance neuro-auditory restoration research toward a human recombinant screening assay.

## Data Availability

No new data were created or analyzed in this study. Data sharing is not applicable to this article.

## Abbreviations

ACh, acetylcholine; AChE, acetylcholine esterase; AT, acoustic trauma; BBB, blood-brain barrier; BLB, blood-labyrinth barrier; HBA, h-bond acceptor; HBD, h-bond donor; HHL, hidden hearing loss; IT, intratympanic; IV, intravenous; LGICs, ligand-gated ion channels; mAChR, muscarinic acetylcholine receptor; MET, mechanoelectrical transduction MOC, medial olivocochlear; MW, molecular weight; nAChR, nicotinic acetylcholine receptor; OHCs, outer hair cells; OW, oval window; PAM, positive allosteric modulator; RB, rotatable bond; RW, round window; TPSA, topological polar surface area.
